# Supplementary material for: Descriptive Sensory Attributes and Volatile Flavor Compounds of Plant-Based Meat Alternatives and Ground Beef
Source: Molecules. 2023 Mar 31;28(7):3151. doi: 10.3390/molecules28073151 (PMC10096055; doi:10.3390/molecules28073151)
Supplement: Supplementary file 1 [file molecules-28-03151-s001.zip › molecules-2287032-supplementary.pdf]

Table S1. Definitions and standard references for descriptive beef flavor and texture attributes where 0 = extremely dry/tough/not detectable and 100 = extremely juicy/tender/intense, from Adhikari et al. (2011) and AMSA (2015).

Attribute	Definition	Reference
Flavor Attributes		
Beef Identity	Amount of beef flavor in a sample	Swanson's Beef Broth = 30<br>80% Lean Ground Beef = 50<br>Beef brisket = 75
Bitter	The fundamental taste factor associated with a caffeine solution	0.01% caffeine solution = 15<br>0.02% caffeine solution = 25
Bloody/Serumy	The aromatics associated with blood on cooked meat products. Closely related to metallic	USDA Choice Strip Steak cooked to 60°C = 40
Brown	The flavor associated with grilled beef; caramelized.	Beef suet = 60<br>Rare strip steak = low<br>Well done strip steak = high
Buttery	Sweet, dairy-like aromatic associated with natural butter.	Land O' Lakes Unsalted Butter tasted = 40
Fat-Like	The aromatics associated with cooked animal fat	90% Lean Ground Beef = 30<br>70% Lean Ground Beef = 60<br>Hillshire Farm's Lit'l Smokies = 44
Liver-like	The aromatics associated with cooked organ meat/liver	Flat iron steak = 20<br>Calf liver = 90
Metallic	The impression of slightly oxidized metals such as iron, copper, and silver spoons	0.10% potassium chloride solution = 10<br>USDA Choice Strip Steak cooked to 60°C = 25<br>Dole Canned Pineapple Juice = 38
Musty/Earthy	Musty, sweet, decaying vegetation	Mushroom = 20
Nutty	Nutty characteristics are: sweet, oily, light brown, slightly musty and/or buttery, earthy, woody, astringent, bitter, etc.	Shelled Walnuts = 43
Overall Sweet	Combination of sweet taste and sweet aromatics; the aromatics associated with the impression of sweet	Post Shredded Wheat = 10
Oxidized	The aromatics associated with oxidized fats and oils. These aromatics may include cardboard, painty, varnish and fishy.	Hillshire Farm's Beef Lit'l Smokies = 20<br>Wesson vegetable oil microwaved for 3 min<br>Wesson vegetable oil microwaved for 5 min
Roasted	The flavor associated with roasted beef.	80% Lean Ground Chuck = 67<br>Chuck Roast, Roasted = 45
Smokey-Charcoal	An aromatic associated with meat juices and fat dripping on hot coats which can be acrid, sour, burned, etc.	Wright's Natural Hickory Seasoning in water = 60
Salty	The fundamental taste factor associated with a sodium chloride solution	0.15% sodium chloride solution = 10

		0.25% sodium chloride solution = 45
Sour	The fundamental taste factor associated with citric acid.	0.015% citric acid solution = 10
		0.25% citric acid solution = 25
Umami	Flat, salty, somewhat brothy. That taste of glutamate, salts of amino acids and other molecules called nucleotides	Unsalted beef broth = 30
		0.035% Accent Flavor Enhancer Solution = 50
Texture Attributes		
Cohesiveness	Amount which sample deforms rather than crumbles, cracks, or deforms.	Corn Muffin = 6
		Yellow American Cheese = 30
		Soft Pretzel = 53
		Starburst = 83
Hardness	Force required to achieve deformation of patty i.e., cut through with incisors or compress with molars.	Cream cheese = 6
		Yellow American Cheese = 30
		Frankfurter = 46
		Cocktail Peanuts = 60
		Life Saver = 96
Particle Size	Size of particles after 5 chews.	Small pearly tapioca = 26
		Boba tea tapioca = 53
Juiciness	Amount of perceived juice released from patty during mastication.	Carrot = 56
		Mushroom = 66
		Cucumber = 80
		Apple = 90
		Watermelon = 100

Table S2. Retention time, fragment ions, and odor descriptors of volatile compounds identified in three plant-based meat alternatives and two lean levels of ground beef.

Volatile Compound (ng/g)	Retention Time[1]	Fragment Ions[2]	Odor/Aroma Descriptor[3]
Alcohols			
Ethanol	2.32	45, 46, 47	Medicinal, alcoholic
1-Hexanol	13.17	56, 55, 69	Green, fruity, apple skin
1-Octanol	20.30	55, 56, 84	Fatty, waxy, citrus, oily, walnut, chemical
1-Octen-3-ol	17.22	72, 83	Mushroom, earthy, fungal
1-Pentanol	9.57	55, 57, 70	Fusel oil, balsamic, bread, fermented
1-Penten-3-ol	6.33	57, 41, 39	Bitter, mild green
2,3-Butanediol	9.54	45, 43, 57	Creamy
Aldehydes			
Decanal	23.99	57, 82, 112	Waxy, orange, citrus
Dodecanal	26.50	57, 82, 95	Soapy, waxy, citrus
Heptanal	14.44	96, 81, 55	Oily, fatty, rancid
Hexanal	10.74	56, 72, 82	Green, fatty, warmed-over, tallow
Nonanal	21.48	96, 82, 114	Citrus, fatty, green, waxy
Octanal	18.06	84, 110, 100	Harsh, fatty, orange, soapy, green
Pentanal	7.26	58, 55, 86	Almond, malt, pungent, acrid, winey, fermented
2-Undecenal	26.24	70, 83, 57	
(E,E)-2,4-Decadienal	25.44	81, 152, 67	Deep fat, fried, chicken flavor at 10ppm
Carboxylic acids			
Acetic acid	4.69	60, 45, 43	Sour, vinegar
Butanoic acid	9.64	60, 73, 55	Sweaty, rancid
Heptanoic acid	19.63	73, 87, 101	Cheesy, fruity, dirty
Nonanoic acid	24.88	129, 115, 73	Fatty, oily, nutty
Octanoic acid	23.05	73, 101, 115	Unpleasant, burning, rancid, fruity-acid, sour
Esters			
Methyl butyrate	7.98	74, 57, 71	Apple, sweet, nutty, creamy
Methyl hexanoate	15.13	74, 87, 99	Ether-like, caproic
Methyl nonanoate	23.91	74, 87, 141	Wine, coconut, sweet
Methyl octanoate	21.89	74, 87, 127	Winey, fruity, orange, oily
Methyl propionate	4.90	57, 59, 80	Fruity, rum, sweet
Furans			
Furfural	12.06	95, 96, 67	Brown, sweet, woody, bready, nutty, caramellic
2-Furan methanol	12.83	98, 81, 69	Warm, oily, burnt, cooked sugar
2-Pentyl furan	17.63	81, 91, 53	Green bean, butter, caramel
2-Methyl-3-furanthiol	13.40	114, 85, 71	Meaty, roasted meat, boiled meat
5-Methyl furfural	17.22	110, 109, 53	Sweet, spiced, caramel-like
Hydrocarbons			

Benzene	6.40	78, 79, 77	Pleasant, distinct
Decane	17.84	57, 71, 142	-
Ethylbenzene	13.18	106, 91, 77	-
Nonane	14.39	57, 85, 71	-
Octane	10.66	114, 85, 43	-
Styrene	13.18	106, 91, 105	Sweet
Tetradecane	25.34	57, 71, 85	Alkane
Toluene	9.75	92, 91, 65	Gasoline, paint thinner
p-Xylene	14.38	91, 106, 105	-
1-Octene	9.57	70, 83, 112	-
Ketones			
Acetoin	7.76	88, 73, 55	Buttery, creamy, fresh
2,3-Butanedione	4.42	86, 87, 56	Buttery
2-Butanone	4.56	72, 57, 43	Chemical, fruity-green
2-Heptanone	13.94	58, 71, 114	Fruity, spicy, cinnamon, cheesy
2-Pentanone	6.91	86, 71, 58	Sweet, fruity, banana
2-Propanone	2.87	43, 58, 42	Pungent, acetone, chemical, earthy
Lactones			
Butyrolactone	15.03	85, 56, 55	Milky, creamy
Oxolanes			
2-Methyltetrahydro-3-furanone	11.16	100, 72, 43	Coffee, bready
Pyrazines			
Methylpyrazine	11.85	94, 67, 53	Nutty, chocolate, roasted
Trimethylpyrazine		122, 81, 54	Musty, potato, roasted, earthy
2,5-Dimethylpyrazine	15.05	108, 81	Musty, potato, cocoa, baked, meaty
2-Ethyl-3,5/6-dimethylpyrazine	20.50	135, 136, 108	Roasted nuts, coffee, chocolate, roasted
Pyrroles			
2-Acetylpyrrole	20.32	94, 109, 66	Bread, walnut, licorice
Strecker Aldehydes			
Acetaldehyde	2.07	44, 43, 45	Fresh, green
Benzaldehyde	17.02	106, 77, 51	Almond oil, nutty, woody
Butyraldehyde	4.55	72, 76, 57	Musty, fusel, bready, yeasty
Methional	14.78	104, 48, 76	Roasted, chocolate, potato
Phenylacetaldehyde	19.81	91, 92, 65	Rosy, floral
2-Methylbutanal	6.33	57, 86, 71	Malty, green, musty, fruity, brothy, meaty
3-Methylbutanal	6.14	86, 71, 58	Malty, brothy, meaty
Sulfides			
Carbon disulfide	3.63	76, 77, 88	Cabbage, sulfurous
Diallyl sulfide	13.21	45, 73, 114	Garlic, alliaceous
Dimethyl disulfide	8.51	94, 79, 45	Onion, sulfurous, unpleasant
Dimethyl sulfide	3.27	62, 47, 45	Asparagus, putrid
Furfuryl sulfide	26.50	194, 81, 53	Cooked onion/garlic, pungent

Terpenes			
α-Pinene	15.89	93, 91, 92	Piney, citrus, turpentine
d-Limonene	19.23	68, 93, 136	Lemon, citrus, fresh
Thiols			
Methanethiol	2.32	48, 47, 45	Vegetable oil, alliaceous, eggy, creamy, sulfuric
Thiophenes			
3-Methyl thiophene	10.40	97, 98, 45	Sweet, meaty

[1] Retention time of a volatile compound eluting through a VF5-ms column; [2] Ions produced at 70 eV electron ionization; [3] Reported odor/aroma descriptor in the literature [3,8,9,10,20,29,30].
